# Intracranial Pressure Elevation 24 h after Ischemic Stroke in Aged Rats Is Prevented by Early, Short Hypothermia Treatment

**DOI:** 10.3389/fnagi.2016.00124

**Published:** 2016-05-27

**Authors:** Lucy A. Murtha, Daniel J. Beard, Julia T. Bourke, Debbie Pepperall, Damian D. McLeod, Neil J. Spratt

**Affiliations:** Translational Stroke Research Laboratory, Faculty of Health and Medicine, School of Biomedical Sciences and Pharmacy, Hunter Medical Research Institute and The University of NewcastleCallaghan, NSW, Australia

**Keywords:** ischemic stroke, intracranial pressure, therapeutic hypothermia, aged rats, middle cerebral artery occlusion, cerebral edema

## Abstract

Stroke is predominantly a senescent disease, yet most preclinical studies investigate treatment in young animals. We recently demonstrated that short-duration hypothermia-treatment completely prevented the dramatic intracranial pressure (ICP) rise seen post-stroke in young rats. Here, our aim was to investigate whether a similar ICP rise occurs in aged rats and to determine whether short-duration hypothermia is an effective treatment in aged animals. Experimental middle cerebral artery occlusion (MCAo-3 h occlusion) was performed on male Wistar rats aged 19–20 months. At 1 h after stroke-onset, rats were randomized to 2.5 h hypothermia-treatment (32.5°C) or normothermia (37°C). ICP was monitored at baseline, for 3.5 h post-occlusion, and at 24 h post-stroke. Infarct and edema volumes were calculated from histology. Baseline pre-stroke ICP was 11.2 ± 3.3 mmHg across all animals. Twenty-four hours post-stroke, ICP was significantly higher in normothermic animals compared to hypothermia-treated animals (27.4 ± 18.2 mmHg vs. 8.0 ± 5.0 mmHg, *p* = 0.03). Infarct and edema volumes were not significantly different between groups. These data demonstrate ICP may also increase 24 h post-stroke in aged rats, and that short-duration hypothermia treatment has a profound and sustained preventative effect. These findings may have important implications for the use of hypothermia in clinical trials of aged stroke patients.

## Introduction

Age is the most important independent non-modifiable risk factor for ischemic stroke, and with an aging population, the burden of stroke is expected to increase dramatically. The difficulty, expense and mortality rate in older animals has resulted in very few aged animal stroke studies being conducted, despite the STAIR criteria suggesting the use of aged animals following initial evaluations in young and healthy animals (Fisher et al., 2009). Such testing is a key part of the strategy for successful translation from bench to bedside (O’Collins et al., 2006). Previous investigations by our group have demonstrated that early treatment with short-duration hypothermia has a preventative effect on intracranial pressure (ICP) elevation, 24 h post-stroke in young rats (Murtha et al., 2014a, 2015). In this short report we aimed to: (1) Determine whether ICP elevation occurred post-stroke in aged rats, and (2) Determine whether a short duration of hypothermia treatment prevented this ICP elevation post-stroke in aged rats.

## Materials and Methods

### Animals

Experiments were performed on male outbred Wistar rats, aged 19–20 months (*n* = 16; 623–952 g). This study was carried out in accordance with the Australian Code for the Care and Use of Animals for Scientific Purposes (National Health and Medical Research Council, 2013) and was approved by the University of Newcastle’s Animal Care and Ethics Committee (A.2011.112).

### Experimental Protocol

Experimental protocols were conducted according to our established techniques in young and aged animals (McLeod et al., 2011, 2013, 2015; Murtha et al., 2014a,b, 2015; Beard et al., 2015). Following anesthetic induction, a right femoral arterial catheter was inserted for arterial blood pressure (BP) monitoring. A laser Doppler probe and fiber-optic pressure catheter were inserted for monitoring of tissue perfusion and ICP, respectively, and experimental stroke was performed (3 h intraluminal middle cerebral artery occlusion-MCAo). Following 1 h of MCAo, rats were randomly assigned to 2.5 h of hypothermia-treatment (32.5°C) or normothermia (37°C). Animals were then recovered from anesthesia. At 24 h following stroke, neurological deficit scores were measured followed by 1 h of ICP and BP monitoring. Brains were removed for histology.

### Anesthesia and Physiological Monitoring

Rats were anesthetized in isoflurane (5% induction, 1.5–2.0% maintenance) in 60:40% N_2_:O_2_ and spontaneously breathed through a low dead-space nose cone. Core temperature was maintained and monitored via a thermocouple rectal probe and warming plate. Surgical incision sites were injected with subcutaneous Bupivicaine (2 mg/kg 0.05%; Pfizer, Sydney, NSW, Australia). A single subcutaneous injection of Atropine (0.05 mg/kg) was administered to reduce respiratory secretions during surgery. Arterial blood pressure (BP), cerebral perfusion pressure (CPP), respiratory rate (RR), heart rate (HR) and oxygen saturation (SpO_2_) were continuously monitored throughout surgery, as previously described (Murtha et al., 2014a, 2015). Prior to recovery, animals were injected with 0.5 mL subcutaneous saline to aide in rehydration. Rats were returned to cages placed half over a heat mat with free access to food and water. All physiological monitoring equipment was reconnected and a left femoral artery catheter inserted for monitoring at 24 h post-stroke.

### Intracranial Pressure and Laser Doppler Measurement

Cranial surgery was performed according to previously described methods (Murtha et al., 2012). Briefly, the ICP probe (SAMBA Sensors, Gothenburg, Sweden) was inserted epidurally into a hollow, saline-filled, polyether ether ketone (PEEK) screw placed 2 mm lateral and 2 mm posterior from Bregma in the left parietal bone. The laser Doppler probe (Moor Instruments, UK) was inserted into a second hollow PEEK screw placed 5 mm lateral and 2 mm posterior from Bregma in the right parietal bone. The screws were secured with dental cement and an airtight seal was created around each probe using a caulking material (Silagum, Gunz Dental, Sydney, NSW, Australia). Laser Doppler flow (LDF) and ICP was measured at baseline and throughout stroke surgery. Both probes were removed prior to recovery, and the screws sealed with Silagum. The ICP probe was reinserted and sealed in place for 24 h ICP monitoring.

### Experimental Stroke and Hypothermia-Treatment

Stroke surgery was performed using a silicon-tipped monofilament (3.0 mm long × 0.4 mm wide tip) inserted to the base of the right middle cerebral artery via the external carotid artery, as previously described (Spratt et al., 2006). Following 3 h of MCAo, the thread was retracted to the base of the external carotid artery. Occlusion and reperfusion of the middle cerebral artery was confirmed by a respective drop and increase in LDF. One hour after occlusion, animals were randomized to 2.5 h hypothermia-treatment or normothermia under anesthesia. Hypothermia animals were cooled to 32.5°C by evaporative cooling using 70% ethanol spray, a fan at the scapula region of the back, and the reduction of the heat mat temperature to 32.5°C. Normothermia animals were kept at 37°C. Animals were recovered immediately following hypothermia/normothermia in a cage placed half over a heat mat, to allow self-thermoregulation. Neurological deficit testing was performed prior to 24 h physiological monitoring. The forelimb flexion, lateral push and torso twist tests were performed by an assessor blinded to treatment allocation. A total neurological deficit score was given out of 6 where a higher score equated to a larger deficit (Murtha et al., 2014a).

### Histological Analysis

Following the 24 h ICP monitoring, rats were transcardially perfused with saline followed by 4% paraformaldehyde in 0.2 M phosphate buffer. Brains were fixed in-skull in neutral-buffered formalin before processing for hematoxylin and eosin staining. Images were scanned on a high resolution digital scanner (Aperio, Vista, CA, USA) before hemisphere and infarct volumes were traced by an investigator blind to treatment-allocation. Infarct volume was corrected for edema using the formula: corrected infarct volume (mm^3^) = infarct volume × (contralateral volume/ipsilateral volume). Edema was calculated by infarct volume minus corrected infarct volume (Murtha et al., 2015).

### Exclusion Criteria and Statistical Analysis

Subarachnoid hemorrhage, equipment malfunction, and lack of infarct were pre-specified exclusion criteria. A sample size calculation (alpha 0.05, power 0.8) for change in ICP from baseline to 24 h (ΔICP) was performed based on our previously published ICP data in young Wistar rats (Murtha et al., 2015; *n* = 3/group). However, we used a minimum sample size of *n* = 5 to allow for outlier effects and a potentially smaller effect size. Two-tailed Student’s *t*-tests were used to compare differences between the treatment groups at particular time points (unpaired) or between time points within treatment groups (paired). Mann-Whitney *U*-tests were performed to compare neurological deficit scores between treatment groups. Significance was accepted at *p* < 0.05. Data are presented as mean ± standard deviation (SD). Neurological deficit scores are presented as median (25th–75th interquartile range).

## Results

Two animals were excluded prior to surgical intervention: facial tumor (16 months) and unexpected death (19 months). Three animals were excluded following surgical intervention: equipment malfunction (prior to randomization); lack of infarct (normothermia); the presence of blood at the carotid canal extending into the subarachnoid space (normothermia). This hemorrhage was likely due to calcification of the carotid canal causing friction on occluding thread insertion and consequent rupture of the arterial wall. Eleven animals were included in this study.

**Figure 1:**
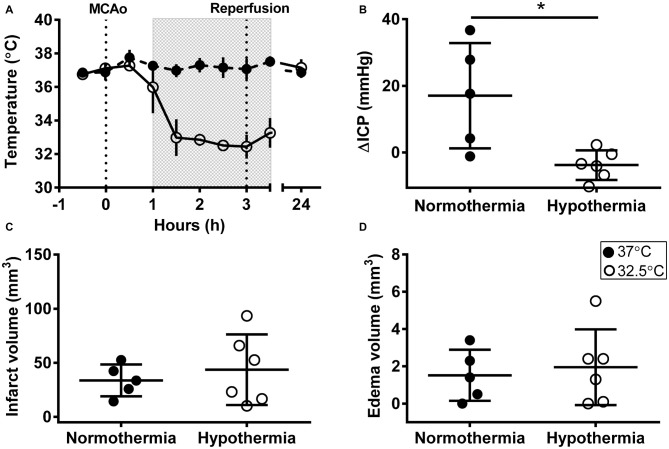
**Intracranial pressure (ICP) elevation after experimental stroke was prevented by early, short-duration hypothermia treatment. (A)** Rectal temperature during middle cerebral artery occlusion (MCAo), and at 24 h post-MCAo, shaded box indicates hypothermia-treatment. **(B)** Change in ICP (ΔICP) from baseline to 24 h post-stroke. **(C)** Infarct volume (corrected for edema) calculated from histological analysis. **(D)** Edema volume calculated from infarct volume minus corrected infarct volume. Individual data ± SD. *p < 0.05. Normothermia (37°C; closed circles; *n* = 5); hypothermia-treated (32.5°C; open circles; shaded box; *n* = 6).

Mean temperature was 32.7 ± 0.7°C during hypothermia treatment and 37.1 ± 0.4°C during the equivalent normothermia period; Figure 1A. Physiological variables are presented in Table 1. ICPs were 10.4 ± 3.6 mmHg and 11.8 ± 3.1 mmHg at baseline (non-significant), and 27.4 ± 18.2 mmHg and 8.0 ± 5.0 mmHg at 24 h (*p* = 0.03) in the normothermic and hypothermia-treated groups, respectively. ΔICP was significantly higher in the normothermic group vs. the hypothermia-treated group (17.0 ± 15.8 mmHg vs. −3.8 ± 4.4 mmHg, *p* = 0.01); Figure 1B.

**Table 1:** **Physiological variables**.

	Baseline		24 h	
	Normothermia	Hypothermia	Normothermia	Hypothermia
BP (mmHg)	89.1 ± 11.8	84.7 ± 8.9	82.3 ± 12.8	82.5 ± 11.2
CPP (mmHg)	79.7 ± 10.3	71.9 ± 7.9	54.9 ± 29.9	73.5 ± 12.1
RR (BPM)	42.1 ± 13.0	58.0 ± 5.5*	48.3 ± 7.4	52.1 ± 10.3
HR (BPM)	333.4 ± 35.5	326.0 ± 28.0	342.6 ± 39.1	339.2 ± 35.0
SpO_2_ (%)	98.6 ± 1.6	95.0 ± 4.8	97.7 ± 1.4	99.0 ± 1.5
Temp. (°C)	36.9 ± 0.2	36.8 ± 0.1	37.5 ± 0.3	37.2 ± 0.5

BP, mean arterial blood pressure; BPM, breaths (RR) or beats (HR) per minute; CPP, cerebral perfusion pressure; HR, heart rate; RR, respiratory rate; Temp., temperature; SpO_2_, oxygen saturation. *p < 0.05 for unpaired t-test between normothermia and hypothermia-treated group at baseline—presented for illustrative purposes only, uncorrected for multiple comparisons.

**Figure 2:**
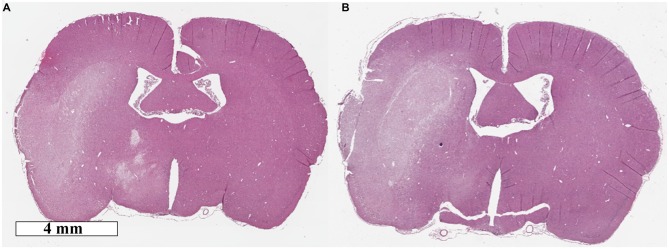
**Representative hematoxolin and eosin images of (A) normothermia and (B) hypothermia-treated animals**.

There was no significant difference in infarct volume between normothermic, 33.7 ± 14.7 mm^3^, and hypothermia-treated groups, 43.6 ± 32.6 mm^3^, *p* = 0.55; Figure 1C. Representative images are presented in Figure 2. Edema volume was small in both groups, 1.5 ± 1.4 mm^3^ and 2.0 ± 2.0 mm^3^, in the normothermic and hypothermia-treated groups, respectively; *p* = 0.70; Figure 1D. No significant difference in neurological deficit scores were seen between the normothermia group [median score 2.0 (0.5–2.5)] and the hypothermia-treated group [median score 2.0 (1.0–3.0)], *p* = 0.61.

## Discussion

In this study, we have shown that the newly discovered phenomenon of ICP elevation at 24 h following minor stroke is also found in most aged animals. Second, the dramatic preventative effect of short-duration hypothermia on ICP elevation is also seen in aged animals. It introduces the possibility that: (1) aged patients may have a similar ICP response following ischemic stroke, and (2) short-duration cooling may prevent ICP elevation in aged stroke patients.

Normal human physiological ICP has been shown to change with age (Dunn, 2002), however our knowledge of ICP changes in the aged stroke population is limited. A study by Schwab et al. (1996) demonstrated in a small cohort of large hemispheric stroke patients that maximum ICP values were considerably higher in patients who subsequently died, than in surviving patients (42 vs. 28 mmHg). They found no correlation between patient age and initial ICP values, however correlation between age and maximum ICP values was not reported. It is important to note that these data applied to patients with large infarct and cerebral edema volumes with a mean ICP device insertion of 2.8 ± 1.5 days post stroke-onset. Conversely, our studies in young and aged rats demonstrated ICP elevation in animals with minor infarct and minimal edema volumes, with a peak around 24 h post stroke-onset (Murtha et al., 2014a, 2015). It is likely that two different ICP elevation mechanisms are at play here. Kotwica et al. (1991) noted that in animals with small-medium strokes ICP peaked 24 h after stroke, however, in animals with large volumes of infarct and edema, a second ICP peak was observed after 3 to 4 days. We believe the initial peak in animals with minor stroke is an edema-independent mechanism of ICP elevation. Given the invasiveness of current monitoring equipment, ICP is only measured in patients with large infarct and edema volumes, thus it is possible that initial 24 h peaks are missed in those with smaller strokes. Interestingly, in the current study, two of the five aged animals in the normothermia group had no biologically significant elevation in ICP. This is in contrast to the young animals in which ICP elevation has been near universal (only three of 30 cumulative young animals had an ICP elevation < 10 mmHg; Murtha et al., 2014a, 2015). Even if ICP elevation occurs only in a proportion of aged patients, we believe the concept of ICP elevation following minor stroke warrants further investigation.

Short-duration hypothermia, administered 1 h after stroke in aged animals, completely prevented ICP elevation at 24 h. This study supports the findings from our previous studies of a profound and sustained ICP prevention with short-duration hypothermia in young animals (Murtha et al., 2014a, 2015). Therapeutic hypothermia has long been known to have a neuroprotective effect following global ischemic brain injury—post cardiac arrest (Bernard et al., 2002), and in neonatal hypoxia ischemia (Shankaran et al., 2005). It has also been used as an effective treatment in reducing ICP following traumatic brain injury and hepatic encephalopathy (Jalan et al., 1999; Tokutomi et al., 2003, 2009). Clinical application and feasibility in stroke patients, however, have been hampered by the logistic challenges of long duration cooling in humans (24–48 h) and concerns about rebound ICP complications during rewarming (Schwab et al., 1998, 2001). Few studies have investigated hypothermia after experimental stroke in aged animals, and those that have (long duration gaseous hypothermia, 24–48 h) demonstrated temporary neuroprotection (Florian et al., 2008; Joseph et al., 2012; Sandu et al., 2016; Vintilescu et al., 2016). ICP, and thus rebound elevations during rewarming, however, were not measured in these studies. The majority of experimental animal stroke studies have been performed in young animals and have shown clear neuroprotective benefit with short duration hypothermia (<6 h; van der Worp et al., 2007; Dumitrascu et al., 2016). Furthermore, when a short duration of mild or moderate hypothermia is administered to young or aged rats many hours before ICP begins to rise, there is a sustained preventative effect, without rebound ICP elevation (Murtha et al., 2014a, 2015). Further experimental studies are clearly needed prior to clinical translation, i.e., dosage, time-window and large animal studies. However, if a short period of cooling by 2–5°C is all that is required for sustained ICP prevention, then clinical translation may lead to a quicker, easier and potentially safer treatment than the current long-duration hypothermia protocols.

ICP was chosen as the primary outcome for the current study, rather than infarct volume, to replicate our previous studies in young animals. Our sample size calculation was therefore based on the ICP data from our young animal studies, and was not powered to show differences in infarct volume. The random distribution of variable infarct volumes in a small study may account for the lack of significance for hypothermic neuroprotection. Our previous work has shown that inadvertent occlusion of the anterior choroidal artery is a major source of the variability seen in the filament MCAo model (McLeod et al., 2013). In particular it may account for the dichotomous infarct volume distribution often seen, and which was apparent in the hypothermia group in the current study. Importantly, ICP elevation was completely prevented by hypothermia-treatment even in the animals with larger infarct volumes.

In conclusion, this study has confirmed that the edema-independent ICP rise following minor stroke that was seen in young animals, also occurs in aged animals. Furthermore, we have confirmed that this ICP elevation is prevented by early short-duration hypothermia treatment. Larger scale studies will be required to determine whether this effect on ICP results in smaller infarcts and better neurological recovery. However, these findings, coupled with our recent data showing effects of ICP elevation on collateral blood flow (Beard et al., 2015) indicate that this newly described pathophysiological phenomenon may be of significant potential clinical importance in aged patients.

## Author Contributions

LAM, DDM, JTB and NJS were involved in the conception and design of the experiments. LAM performed the surgical and experimental components of the study, analyzed the data, including statistical analysis, and drafted the manuscript. Histological analysis and neurological testing was performed by DP. LAM, DDM, DJB and NJS discussed and interpreted the results and reviewed and edited the manuscript.

## Funding

This work was supported by a NHMRC Project Grant, APP1033461, and research support from the Hunter Medical Research Institute from funds donated by the Greater Building Society. LAM was supported by an Emlyn and Jennie Thomas Postgraduate Scholarship. NJS received a NHMRC career development fellowship, APP1035465.

## Conflict of Interest Statement

The authors declare that the research was conducted in the absence of any commercial or financial relationships that could be construed as a potential conflict of interest.

## Abbreviations

BP: mean arterial blood pressure
BPM: breaths (RR) or beats (HR) per minute
CPP: cerebral perfusion pressure
HR: heart rate
ICP: intracranial pressure
LDF: laser Doppler flow
MCAo: middle cerebral artery occlusion
RR: respiratory rate
SD: standard deviation
SpO_2_: oxygen saturation
Temp.: temperature.
